# Emerging mechanisms of immunotherapy resistance in sarcomas

**DOI:** 10.20517/cdr.2021.111

**Published:** 2022-03-05

**Authors:** Vaia Florou, Breelyn A. Wilky

**Affiliations:** ^1^Department of Medicine, Huntsman Cancer Institute at University of Utah, Salt Lake City, UT 84112, USA.; ^2^Department of Medicine, University of Colorado School of Medicine, Aurora, CO 80045, USA.

**Keywords:** Sarcoma, immune therapy, immune resistance, immunogenicity, chemotherapy, clinical trials

## Abstract

Sarcomas are a heterogeneous group of over 150 mesenchymal neoplasms of bone and soft tissue. Clinical prognosis remains poor in the metastatic and refractory setting, despite treatment with traditional chemotherapies. A subset of sarcoma patients can exhibit remarkable responses to novel immune therapies; however, most patients will not respond. Emerging data from genetic and transcriptomic datasets suggests that patients who are resistant to checkpoint inhibitor monotherapy may have low expression of immune-related genes, suggesting that the sarcoma was not sufficiently immunogenic to trigger or maintain an immune response to generate tumor-specific immune effector cells. In this review, we discuss the emerging data surrounding potential mechanisms of resistance, including various biomarkers explored in clinical trials of immune therapy for sarcomas. We also review future directions in clinical trials that are focused on boosting tumor immunogenicity to improve the activity of checkpoint inhibitors, as well as adoptive cellular therapy approaches to bypass deficiencies in neoantigens or antigen presentation.

## INTRODUCTION

Sarcomas consist of a rare and heterogeneous group of bone and soft tissue malignancies with more than 150 different histologic subtypes by the latest World Health Organization classification^[1]^. While localized sarcomas can be cured with aggressive surgery and radiation treatments, in general, metastatic and refractory sarcomas are incurable with cytotoxic chemotherapies with a median overall survival of 12-18 months^[2]^. Sarcomas have long been theorized to be recognizable by the immune system (tumor immunogenicity), dating back to early observations by Dr. William Coley^[3]^ in the 1890s, who noted that some patients with sarcoma had tumor regressions following bacterial infections. With the modern revolution in cancer immunotherapy, interest in exploring novel treatment approaches such as immune checkpoint inhibitors and adoptive cellular therapies has rebounded for sarcomas, with numerous clinical trials being conducted in a wide variety of sarcoma subtypes (See Table 1 for representative trials). While there have been a few promising signals of activity, such as engineered T cell therapy targeting cancer/testis antigens in synovial sarcomas, and remarkable responses to checkpoint inhibitors in alveolar soft part sarcoma (ASPS) and cutaneous angiosarcomas, responses to immunotherapy for most sarcoma subtypes have remained modest^[4-10]^. To further improve the activity of immune-modulating therapies in sarcomas, it is critical that we learn from the extensive work in other cancers aimed to explore mechanisms of response and resistance, and better understand the barriers existing in the sarcoma tumor and immune microenvironment. In this review, we will explore the emerging mechanisms of immune evasion in sarcoma and highlight ongoing studies in immunotherapeutics aimed to reverse this resistance.

**Table 1:** Selected immune checkpoint inhibitor combination clinical trials for sarcomas by mechanism of resistance

**Resistance mechanism**	**Agents**	**Ref. (if data available)**	**NCT# (if ongoing)**
**Suppressive immune phenotypes (TAMs, Tregs)**	Cyclophosphamide/pembrolizumab	[65]	**-**
	DCC-3014 (CSF1R inhibitor)/avelumab	[64,65,90]	**NCT04242238**
**Microenvironment**	Axitinib/Pembrolizumab	[4]	**-**
	Sunitinib/Nivolumab	[36]	**-**
	Apatinib/Camrelizumab	[37]	**-**
	Cabozantinib/Nivolumab +/- Ipilimumab	-	**NCT04339738** **NCT04551430** **NCT05019703**
**Tumor immunogenicity**	Radiation +/- pembrolizumab	-	**NCT02301039**
	**Cytokines:**		
	NKTR-214/nivolumab	[75]	**-**
	IFN-gamma/pembrolizumab	-	**NCT03063632**
	**Oncolytic viruses:**		
	Talimogene laherparepvec (TVEC) /pembrolizumab	[91]	**-**
	**Chemotherapy:**		
	Doxorubicin/pembrolizumab	[32,87]	**-**
	Doxorubicin/zalifrelimab/balstilimab	-	**NCT04028063**
	Doxorubicin/ifosfamide/PD-1 blockade	-	**NCT04356872**
		-	**NCT04606108**
	Gemcitabine/docetaxel/retifanlimab	-	**NCT04577014**
	Gemcitabine/pembrolizumab	-	**NCT03123276**
	Gemcitabine/docetaxel/doxorubicin/nivolumab	-	**NCT04535713**
	Trabectedin/ipilimumab/nivolumab		**NCT03138161**
	Eribulin/pembrolizumab	[88,89]	**NCT03899805**

### Overview of immune response and evasion

**Figure 1:**
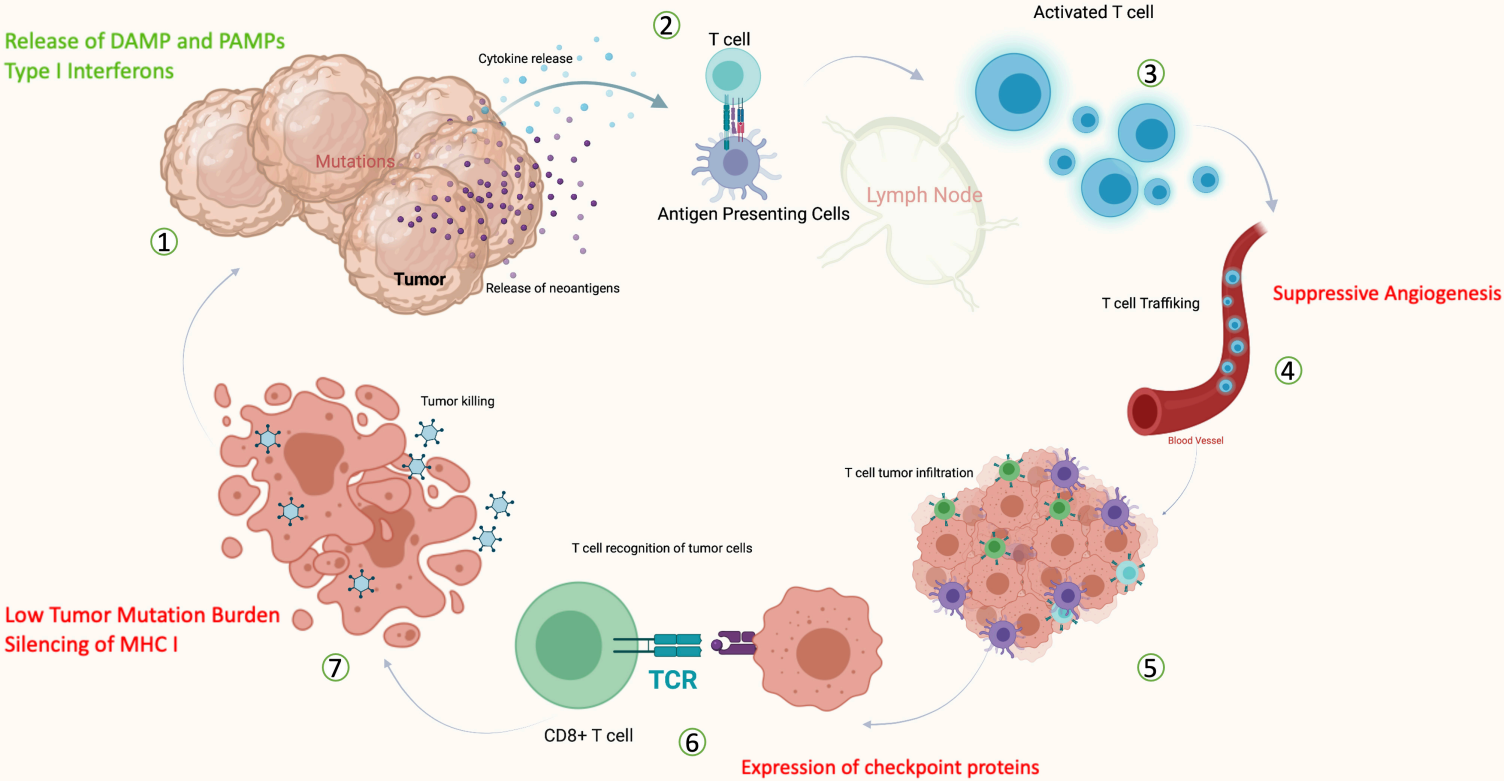
Multiple steps are required to generate immune response against cancer. Inhibitory factors are noted on red and facilitating factors in green. DAMP: Damage-associated molecular patterns; PAMPs: pathogens-associated molecular patterns; MHC-I: major histocompatibility complex class I; TCR: T cell receptor. Created with BioRender.com.

To mount an effective immune response against cancer cells, a sequence of multiple events is required [Figure 1], with aberrations along any of the steps potentially leading to immune evasion and resistance^[11]^. First, the cancer cell must have immunogenic tumor-specific antigens largely resulting from genetic mutations that are processed and presented in conjunction with major histocompatibility complex class I (MHC-I). At these early phases, cancers that lack mutations (such as those driven by translocations), or present weakly immunogenic antigens, or have faulty antigen-processing machinery may fail to elicit an immune response^[11]^. Mutations in antigen presentation machinery components such as deletions in the beta-2 microglobulin subunit of MHC-I, deletions in the primary antigen processing enzymes such as transporter associated with antigen processing, or epigenetic silencing of MHC-I can all stall antigen presentation within the cancer cell^[11]^. Once antigens have been processed and peptides expressed on the surface of the cell, antigen-presenting cells (APCs) such as dendritic cells (DCs) and macrophages must then respond to immunogenic cytokines from the cancer cells to be drawn into the tumor microenvironment (TME) and recognize the antigens^[11]^. Insults that trigger immunogenic cell death of the cancer cells, such as certain types of chemotherapy and radiation, can lead to the production of type I interferons and release of other damage and pattern-associated receptors (DAMPs and PAMPs) that improve APC migration and activation^[12]^. Effective APC surveillance and uptake of antigens can then lead to the priming of tumor-specific T cells in tumor-draining lymph nodes that then expand, traffic, and infiltrate into the tumor beds^[11]^. Alternatively, cytotoxic T cells may directly surveil MHC-I bound antigen on the surface of the cell to trigger activation and T cell-mediated killing. Regardless, at the final stages of the cytotoxic response, the cancer cell: T cell synapse is again modulated by the expression of immune checkpoint proteins such as the programmed cell death protein 1/programmed cell death protein ligand 1 (PD-1/PD-L1) axis, which can abort T cell-mediated killing even in the setting of a robust prior immune response^[11]^.

A major obstacle for both APC and T cell trafficking and function is navigating the suppressive TME and retaining cytotoxic and anti-tumor potential under stress from faulty metabolism, aberrant angiogenesis, and stromal factors that slow or inhibit the infiltration of immune cells into the tumor bed. Both DCs and macrophages are versatile innate immune cells with functional plasticity that is influenced by the TME. Activated and suppressive phenotypes exist in a delicate balance, and with prolonged exposure to the TME, there is a preponderance of suppressive immature DCs and tumor-associated macrophages (TAMs). TAMs have a complex nomenclature, with numerous functionally distinct phenotypes. A common terminology used includes M0, newly recruited monocytes to the TME prior to differentiating to M1/M2; M1, classically activated macrophages, which have anti-tumor properties; and M2, alternatively activated macrophages which have immunosuppressive activity^[13,14]^. Finally, T cells progressing through various activation stages will eventually become exhausted, losing their cytotoxic potential, or skew towards suppressive phenotypes such as T regulatory cells^[15]^.

The landscape of immunotherapies being studied in sarcoma aims to reverse immune evasion by the cancer cells at each of these steps. However, the immune evasion and kill balance is a highly dynamic process that is dependent on numerous factors, including tumor heterogeneity within individual tumors even within individual patients, as well as extensive host factors^[16]^. A variety of modalities ranging from checkpoint inhibitors, adoptive T cell transfer, oncolytic viruses, cancer vaccines, and cytokine modulation have been tested. However, one of the biggest challenges in immunotherapy in general and particularly in sarcomas is understanding the mechanisms of resistance to these therapies at any particular time, to tailor and develop therapies that can overcome the chief resistance mechanisms sequentially, or multifaceted approaches that target multiple steps in the cascade simultaneously. We will now review the emerging data in sarcomas for resistance and how this is impacting the development and efficacy of immunotherapeutic strategies.

## EMERGING MECHANISMS OF RESISTANCE

### Preclinical investigations in murine models

In-depth exploration of the sarcoma immune microenvironment has been limited due to the few numbers of syngeneic murine models with a competent immune system. Additionally, those models that do exist fail to capture the heterogeneity within a large number of sarcoma subtypes, genetic and immune heterogeneity within sarcomas even of the same subtype, and inter-and intratumoral heterogeneity within the same patient. Wisdom *et al.*^[17] ^recently reported immune profiling in a high mutational load murine model of undifferentiated pleomorphic sarcoma (UPS), generated by inducing conditional *Trp53*^fl/fl ^deletions with subsequent injection of the carcinogen 3-methylcholanthrene in the same site. The authors demonstrated resistance to radiation and PD-1 blockade in primary tumors that evolved under immune pressure, compared to transplanted tumors in different mice, which were more readily rejected. As expected, transplanted tumors showed a more inflamed microenvironment with increased activity of suppressive M2 macrophages and activated CD8+ T cells. Treatment with PD-1 blockade further increased CD8+ T cell activity with a decrease in M2 macrophages, which was not recapitulated in the primary tumors^[17]^. However, another study performed similar experiments in the KP model, with conditional *Kras*^G12D ^mutation plus *Tp53 *deletion, comparing the immune infiltrates and response to checkpoint blockade in primary tumors to those transplanted into naïve C57Bl/6 mice. Interestingly, in the setting of a low mutational burden tumor, there was no improvement in immunogenicity despite the transplantation, and despite an increase in immune cell infiltration, there was no improvement in response to checkpoint blockade^[18]^. Finally, a third paper summarized divergent immune landscapes with transplantation of primary KP UPS and rhabdomyosarcoma models into syngeneic recipient mice. Again, a more robust immune infiltration was seen in the recipients, which was associated with higher levels of MHC-I expression^[19]^. Taken together, these papers illustrate that for “cold”, antigen-poor primary sarcomas, recognition of the cells as foreign is critical to mounting an effective immune response. The lack of immunogenic neoantigens or failure of the initial recognition by APCs is likely one of the central mechanisms of resistance to immunotherapy for the majority of sarcomas.

### Global assessment of immunogenicity using immune-related gene expression analysis

With the increase in available transcriptomic data, multiple studies have recently investigated immune-related gene expression signatures in human sarcomas. In an analysis of 213 sarcoma tissue samples from The Cancer Genome Atlas (TCGA), including leiomyosarcoma (LMS), UPS, and dedifferentiated liposarcomas (dLPS), deconvolution analysis showed that the majority of sarcomas lacked high expression of immune-related pathway signatures. However, 20% exhibited an immune-high expression signature which correlated with responses to pembrolizumab monotherapy and was characterized by the presence of tertiary lymphoid structures (TLSs), comprised of CD8+ T cells, B cells, and follicular dendritic cells^[20]^. Additionally, these sarcomas showed high expression of early immune response signatures, including MHC-I. Close to 80% of the sarcomas had a low or heterogeneous immune signature expression, supporting clinical observations of checkpoint inhibitors inducing a response in only about 20% of patients^[4-10]^. A subsequent paper utilized the CIBERSORT algorithm to query microarray expression data in 253 soft tissue sarcomas [synovial sarcoma, myxoid liposarcoma, non-translocation sarcomas with more complex genomic profiles, and gastrointestinal stromal tumor (GIST)] and identified immune signatures that were largely clustered within the histologic subtype. Several potential targets of interest were identified for the various histologies, such as CD40 in GIST and synovial sarcomas, and M0 macrophages were enriched for all subtypes. These signatures had some prognostic implications but were not correlated with any prior immunotherapy exposure^[13]^. A third paper from Hu *et al.*^[21] ^similarly reviewed immune-related expression signatures in 259 patients with soft tissue sarcomas and again demonstrated four unique clusters of immune-related genetic expression and demonstrated associations with prognosis, including overall survival and progression-free survival. The worst prognosis cluster had the lowest immune score, lowest stromal score, and lower expression of a variety of checkpoint proteins. These collective investigations again support that failure to generate an immune response, evidenced by a lack of downstream immune activity, may be central to the resistance of most sarcomas to immune targeting therapies.

### Neoantigens and tumor mutational burden

It has been widely recognized that a high tumor mutational burden (TMB), which can be detected by whole-genome sequencing (WGS), can lead to increased neoantigen expression and can correlate with immunotherapy responses^[22,23]^. Although TMB is not a perfect biomarker, most sarcomas do have a lower TMB than the currently approved tissue agnostic threshold (10 mutations/Mb) that has been shown to predict responses to checkpoint inhibitors^[24]^. However, 13.7% of angiosarcomas, 8.1% of UPS, and 8.2% of malignant peripheral nerve sheath tumors (MPNSTs) were found to have more than 20 mutations/Mb in an analysis of 100,000 different cancers^[24]^, potentially explaining the differential activity of immune checkpoint inhibitors (ICIs) in these subtypes. In contrast, in subtypes where responses to ICIs are poor, like synovial sarcoma and osteosarcoma, only 1% and 0.4% respectively had high TMB greater than 20 mutations/Mb^[24]^. However, TMB does not fully explain the immunogenicity of individual sarcomas with low TMB or for remarkable responses seen in translocation-associated sarcomas such as ASPS^[25]^. Some evidence exists that perhaps genetic abnormalities not picked up with standard WGS, such as fusions without an RNA product, or indels, may help to drive responses. We previously reported whole-exome and RNA sequencing results for a patient with cutaneous angiosarcoma who had a complete response to very low dose cytotoxic T-lymphocyte-associated protein 4 (CTLA-4) inhibition on a phase 1 clinical trial^[5]^. While many cutaneous angiosarcomas have been shown to have high TMB with an ultraviolet radiation damage signature resembling melanoma^[26]^, our patient had a very low TMB at 0.09 mutations/Mb. However, numerous putative fusion transcriptions were identified, including 31 fusions predicted to generate novel protein sequences. Increasingly, epitopes resulting from non-traditional genetic alterations, such as indels, copy number alterations, or single nucleotide variants, have been recognized to be potent antigens, potentially driving responses to immunotherapy^[27-29]^. As shown in other cancers, the quality of an antigen may dominate over the number of predicted antigens, which may not be conserved from patient to patient, and is dependent on the unique characteristics of the individual patient’s MHC alleles.

Finally, there is emerging evidence that particular genetic alterations may profoundly impact oncologic signaling pathways and the development of resistance to immunotherapy. One powerful example is a patient with metastatic leiomyosarcoma who achieved a durable response to checkpoint blockade for more than two years, but then developed oligoprogression in a single metastatic site. Genetic profiling revealed loss of *PTEN *in the progressing lesion, which reduced neoantigen expression and resulting immunogenicity^[30]^. Another well-reported biomarker of immunotherapy resistance is *MDM2 *amplification which has been linked with hyperprogressive disease with ICIs^[31]^. However, liposarcomas that are defined by *MDM2 *amplification can respond to ICIs alone or in combination with chemotherapy^[9,32]^. Further analysis of genetic mutations in correlation with TME features, such as PD-L1 expression and phenotyping of infiltrating lymphocytes, will be critical to better understand the impact of these genetic aberrations on the sarcoma immune microenvironment.

### Tumor microenvironment

Another leading hypothesis for resistance to immune therapies for sarcomas lies in the hostile TME. Sarcomas tend to be large with aberrant angiogenesis, with frequent overexpression of major suppressive cytokines such as vascular endothelial growth factor (VEGF)^[33] ^or transforming growth factor beta 1 (TGFB1)^[34]^. Since sarcomas are, by definition, stromal/mesenchymal neoplasms, many have theorized that suppressive forces from the stroma itself may drive resistance to immune therapy.

VEGF has been implicated in numerous complex mechanisms of resistance, including the promotion of immature and suppressive phenotypes of DCs, TAMs, and T regulatory cells, impairment of T cell trafficking and migration via faulty angiogenesis, and altering of various physiologic processes via maintenance of hypoxia^[35]^. The activity of various tyrosine kinase inhibitors (TKIs) with anti-VEGF activity has led to multiple combination studies with checkpoint blockade in both bone and soft tissue sarcomas^[4,36,37]^. While the mechanisms through which angiogenesis growth factors enhance immunotolerance could be related to increased vascular permeability^[33]^, correlative investigations to identify biomarkers of response and to elucidate resistance to combination anti-VEGF TKI therapy are still under investigation and will be discussed later in this review.

TGFB has also been implicated as a global mediator of resistance to cancer treatment, including immunotherapy^[38]^. TGFB has been shown to directly inhibit T cell function by promoting FOXP3 expression on T cells, impairing natural killer cell function, decreasing MHC class II expression, and promoting suppressive phenotypes of TAMs and neutrophils^[38]^. While the role of TGFB is underexplored in sarcomas, several studies have shown that TGFB-mediated expression of epithelial-mesenchymal transition (EMT)-type transcription factors, including Snails and Twist, promotes invasion and metastasis of osteosarcoma cells^[39]^. Clinical investigations of TGFB inhibition in sarcoma are very limited, with the inclusion of only a few patients in all-comer Phase 1 studies^[40]^. However, Vigil is an autologous tumor cell product where cells are transfected with a plasmid containing the stimulatory cytokine granulocyte-macrophage colony-stimulating factor (GM-CSF), and a short hairpin RNA to knock down furin, an enzyme required for the production of TGFB1 and TGFB2. Initial results of Vigil in patients with Ewing’s sarcoma showed promising results, with a 73% 1-year survival for Vigil-treated patients compared to 23% in a contemporaneous cohort of patients who did not receive Vigil^[41]^. An ongoing Phase 3 clinical trial of Vigil with irinotecan/temozolomide (NCT03495921) aims to confirm the benefit of targeting TGFB in this population, and may support further investigation of targeting this mediator.

An emerging suppressive factor of interest is sialoglycans, which are sialic acid sugar-carrying glycans, and are suggested to mediate regulatory pro-tumor growth functions of the TME and to induce angiogenesis regulated by macrophages^[42,43]^. Sialoglycans are recognized by sialic acid-binding immunoglobulin-like lectins (Siglecs), a family of immunomodulatory receptors that are expressed predominantly on immune cells. Tumor sialoglycans can interact with the Siglecs and may modulate the immune TME and stimulate further Siglec expression on the infiltrating immune cells^[44]^. Each Siglec preferentially recognizes a different type of sialic acid. Siglec-15, in particular, is selectively expressed in myeloid cells and osteoclasts^[45]^. While studies in other types of sarcoma are lacking, preclinical studies on osteosarcoma cell lines and xenograft mouse models discovered that downregulation of Siglec-15 led to decreased tumor proliferation by inducing EMT and promotion of DUSP1^[46]^. Following that finding, an analysis of 36 human osteosarcoma samples also showed that higher expression of Siglec-15 by immunohistochemistry was associated with worse overall survival compared with tumors with low Siglec-15 expression^[46]^. A pan-cancer analysis utilizing the TCGA database, which included sarcoma, showed that upregulation of Siglec-15 correlated with shorter overall survival and progression-free survival (PFS) in the sarcoma cohort^[47]^. Siglec-15 also positively correlated with T-regulatory cells and macrophages in most cancers^[47]^. Thus, it is intriguing to speculate that the negative impact on survival from Siglec-15 expression could result from its immunosuppressive activity in sarcomas.

### Biomarkers of resistance identified in clinical explorations of immunotherapy vaccines

With a central mechanism of resistance in sarcomas appearing to revolve around a lack of neoantigens that can trigger antigen presentation, immune recognition, and adaptive immune responses, it is not surprising that much hope has previously been placed in the use of therapeutic vaccines. Despite extensive efforts, the development of therapeutic vaccines in sarcomas has shown limited efficacy thus far. For example, many pediatric sarcomas express the gangliosides GM2, GD2, and GD3^[48]^, leading to a study of a peptide vaccine targeting these markers in 136 patients with a variety of sarcomas. Despite high expression, the vaccine did not show any benefits in survival despite eliciting a sustained serologic response^[49]^. In other attempts, dendritic cell vaccines were developed using patient-derived dendritic cells loaded with tumor antigens *ex vivo*. Despite the notable serologic response, this strategy has also been unsuccessful; only 1 out of 35 evaluable patients had a partial response^[50]^. The lack of response to vaccines has been attributed to downstream mechanisms of immune suppression, possibly secondary to increased tumor burden^[50]^. Vaccines using peptide-pulse dendritic cells with a tumor antigen have also been used in an attempt to avoid surgical resection of tumors required to isolate tumor lysates. This strategy was also disappointing^[51] ^with no long-lasting responses secondary to the short lifespan of the cells in the circulation. Overall, vaccines are likely to require combination approaches, potentially agents targeting the microenvironment and/or checkpoint blockade to further improve efficacy.

### Immune checkpoint inhibitors

Immune checkpoint inhibitors have transformed the treatment of multiple solid and hematologic malignancies since their initial success in melanoma and non-small cell lung adenocarcinoma. The antibodies currently approved target the inhibitory proteins PD-1, PD-L1, and CTLA-4, and block the suppressive signals towards T cells in the TME. In sarcomas, the success of checkpoint inhibitors alone is limited for most subtypes, with responses ranging consistently less than 20%^[9,52]^. Exceptions to those responses have been observed in a few histologies such as UPS, dLPS, ASPS, and cutaneous angiosarcoma^[4,5,9,52]^. Since ICIs are now the most commonly tested immunotherapy strategy for sarcoma patients, the majority of our knowledge on immunotherapy resistance mechanisms stems from these trial results.

The expression of PD-L1 by cancer cells is an important predictive factor of response to ICIs in many cancer types and is used as a biomarker of favorable results^[53]^. However, in sarcomas, the predictive value of PD-L1 expression for responses to ICIs as well as outcomes has been inconsistent. Overall though, most subtypes show consistently low expression of PD-L1 in the range of 10%-22%^[54-57] ^and higher in certain subtypes such as UPS and ASPS^[4]^.

There is some evidence that the presence of tumor-infiltrating lymphocytes (TILs) and other immune cells within the sarcoma TME may be more predictive of ICI responses compared to PD-L1 expression on cancer cells. The prevalence of TILs in most common sarcoma subtypes has been reported to be as high as 98%^[58,59]^; however, some of these studies are limited in phenotyping to determine the function of visualized cells. It has been observed that tumors that lack TILs are unlikely to respond to ICIs, regardless of PD-L1 expression^[60]^. In the SARC028 trial of pembrolizumab in patients with advanced sarcoma, the objective response rate was only 17.5%^[9]^. Patients who responded to ICIs, albeit few, had significantly higher infiltration of T cells (CD8+, CD3+, PD-1+) and TAMs that were PD-L1 positive at baseline compared to nonresponders^[61]^. Across a variety of numerous soft tissue sarcoma subtypes, UPS was found to have a higher density of TILs with more oligoclonality compared to other subtypes, further highlighting the role of TILs in ICIs responses^[57]^. However, ASPS patients treated with axitinib/pembrolizumab did not show higher TIL infiltration by immunohistochemistry compared to non-ASPS patients, but had universally positive PD-L1 expression, despite the higher response rate^[4]^.

Taking T cell infiltration one step further, TLSs are organized lymphoid aggregates containing CD8+ T cells, B cells, and follicular dendritic cells^[20]^, that influence anti-tumor immunity in cancers, well-described in melanoma and renal cell carcinoma^[62]^. TLSs have been identified in various sarcoma subtypes, including UPS, dLPS, LMS, and rhabdomyosarcomas^[20,63]^, and their presence correlated with response to pembrolizumab monotherapy^[20]^. As such, TLSs may offer a surrogate of an organized and robust anti-tumor immune response and may be used as a biomarker of response to immunotherapy, as explored in a dedicated arm in a larger phase II clinical trial of pembrolizumab and cyclophosphamide in soft tissue sarcomas^[64]^. Out of 240 sarcoma patients screened for the TLS-containing cohort, 35 evaluable TLS-positive sarcoma patients achieved a 30% objective response rate, with 33.3% achieving stable disease. The overall response rate for the unselected patient population in this trial was only 2%^[65]^.

In addition, many soft tissue sarcomas and GIST tumors have a predominant expression of M2 macrophages, the immune-suppressive phenotype^[65]^. Additionally, myeloid-derived suppressor cells (MDSCs), which are regulatory cells promoting tumor growth and immune evasion in the TME, have been shown to correlate with resistance to ICI in patients with melanoma^[66]^. In sarcoma, MDSCs have been reported to correlate with higher stage and metastatic tumor burden, possibly supporting their immunosuppressive role in the sarcoma TME^[67]^. Further evidence to support that was the findings of enhanced responses with ICI treatment in a murine model of rhabdomyosarcoma upon inhibition of the MDSCs trafficking to the TME^[68]^.

### Adoptive cellular therapy

Adoptive cellular therapy aims to bypass the priming and activation of the T cells by providing *ex vivo *activated T cells against previously-identified tumor antigens directly to the patient. Three adoptive cellular therapies under study currently in sarcomas include engineered T cell receptors (TCR), which recognize MHC-I-restricted antigens, Chimeric Antigen Receptor (CAR) T cells which can recognize cell surface antigens, and adoptive transfer of TIL^[69]^. Cellular therapies harnessing engineered TCRs or CARs require antigen recognition in the tumor, preferentially expressed by tumor cells and not by normal cells. New York-esophageal squamous cell carcinoma-1 (NY-ESO-1) and melanoma-associated antigen-A4 (MAGE-A4) are two unique cancer/testis antigens expressed in selected sarcoma subtypes. NY-ESO-1 expression has been observed more frequently in synovial sarcomas (> 60%) and in myxoid liposarcomas (88.2%) and less frequently in pleiomorphic liposarcoma (21.4%), chondrosarcomas (14.3%), osteosarcomas (31.3%), and desmoplastic small round cell tumors (16.7%)^[70,71]^. Similarly, MAGE-A4 has been detected more commonly in synovial sarcomas (82.2%), myxoid liposarcomas (67.7%), osteosarcomas (43.8%), angiosarcomas (41.4%), MPNSTs ( 24.6,%) and chondrosarcomas (21.4%)^[70,71]^.

The responses to engineered T cells specific to NY-ESO-1 in synovial sarcomas are definitely encouraging, with a 50% objective response rate in the first cohort of treated patients^[8]^. A major challenge for TCR-directed therapies, though, is the required human-leukocyte antigen (HLA) compatibility, with currently studied agents restricted to HLA-A*02 subtypes. In the first cohort of patients treated with NY-ESO-1 T cells, 120 patients were screened for HLA subtype and antigen expression to enroll 15 patients^[8]^. An important insight from this study was that contrary to other cancers, loss of antigen expression and loss or mutations of genes mediating antigen presentation, including MHC-I, were not associated with acquired resistance in patients with synovial sarcoma treated with SPEAR T cells targeting NY-ESO-1^[72]^. However, the lymphodepleting chemotherapy regimen is important for the effectiveness of the TCR therapies in sarcomas; it favors the combination of fludarabine and cyclophosphamide and maximizes the engraftment of adoptively transferred T cells via IL-7 and IL-15^[72]^.

CAR T cells provide an alternative to the HLA compatibility challenge that limits TCR therapy. However, they require antigens expressed on the surface of the tumor cells rather than peptide-MHC complexes (intracellular antigens). Extracellular antigens are limited in sarcomas overall, with a few exceptions of GD2 in osteosarcoma, rhabdomyosarcoma, and Ewing’s sarcoma; platelet-derived growth factor receptor α in rhabdomyosarcoma and HER-2 in osteosarcoma^[48,73,74]^. Studies are currently underway to assess the safety and efficacy of these and other therapies, including CAR T cells directed at B7-H3 (NCT04897321), EGFR (NCT03618381), and GD2 (NCT02107963, NCT04539366, NCT03721068, NCT03635632).

Finally, TIL therapy is under early investigation in sarcomas; in this modality, autologous TILs are collected from an individual patient’s tumor and then expanded, and then returned to the patient with lymphodepleting chemotherapy and interleukin-2 (IL-2) therapy. An ongoing clinical trial is exploring the safety and efficacy of this approach (NCT04052334).

### Overcoming resistance: future directions

While immune therapies, including checkpoint inhibitors and adoptive cellular therapies, have had promising activity in a subset of sarcoma patients, it is clear that other strategies will be needed to overcome innate resistance to immune-mediated recognition and kill the majority of sarcomas. Over the past several years, various trials have tested combination therapies aimed at reversing resistance mechanisms at various steps in the cascade, including targeting the suppressive immune microenvironment with metronomic cyclophosphamide^[65]^, or with VEGF blockade^[4,36,37]^. Selected trials representing the scope of previous and ongoing strategies to overcome key potential mechanisms of resistance discussed in this review are listed in Table 1.

While combination VEGF blockade and checkpoint inhibitors have shown activity in ASPS^[4] ^and osteosarcoma^[37]^, outcomes in other soft tissue sarcomas have not been convincingly different from what could be expected from checkpoint inhibitor therapy alone. Ongoing trials are assessing the activity of broader-spectrum anti-VEGF TKIs, such as cabozantinib (NCT04339738, NCT04551430, NCT05019703), which could improve outcomes. Additionally, transcriptomic analysis suggested a subset of sarcomas with an angiogenic signature^[20]^, thus upcoming correlative analysis from previously conducted trials^[4,36] ^may help to identify a subset of sarcomas benefitting from this approach. Despite this, we still must identify novel strategies to address the majority of sarcomas which lack tumor immunogenicity, the ability of the sarcoma cells to induce and maintain an early immune response.

The main combination strategies aimed at inducing tumor immunogenicity include cytokines, radiation, and cytotoxic chemotherapy together with ICIs. A recent trial combined an IL-2 pathway agonist, NKTR-214, with nivolumab in a large phase I/II study for bone and soft tissue sarcomas (NCT03282344). While early results showed modest responses^[75]^, correlative studies presented in the abstract suggested candidate biomarkers, including changes in PD1/PD-L1 expression in tumor and immune cells with treatment, as well as genetic variations apart from TMB that may impact outcomes^[75]^. The full publication is anxiously awaited as this large study may further help elucidate mechanisms of response and resistance. Additionally, Zhang *et al.*^[76]^ showed in a phase 0 trial that systemic use of interferon-gamma could increase MHC-I expression and T-cell infiltration in patients with synovial sarcoma and myxoid round cell liposarcomas, leading to a phase II trial of interferon-gamma with pembrolizumab (NCT03063632). While no results are yet published for this study, this is another unique strategy to target early immune responses and restore immunogenicity in cold sarcomas. Another mechanism to boost tumor necrosis and early immune responses is radiation therapy, and the final results of an ongoing study of preoperative radiation therapy for stage III UPS and dLPS with or without concurrent/adjuvant pembrolizumab (NCT02301039) are likely to contribute greatly to our understanding of the immune impact of this critical component of therapy for newly diagnosed sarcomas.

Finally, we will now discuss the emerging strategy of combining checkpoint blockade with cytotoxic chemotherapy, to induce tumor immunogenicity, neoantigen production, and trigger an early immune response, in hopes of potentiating the downstream activity of ICIs.

### Chemotherapy combinations

One of the most potential inducers of tumor immunogenicity is traditional chemotherapy, with various agents inducing immunogenic cell stress, production of innate immunity attractants including type 1 interferons, and inflammasome induction^[77]^. In particular, doxorubicin has been shown in a variety of preclinical tumor models to potently induce the production of DAMPs and type 1 interferons promoting downstream transcription of interferon-stimulated genes, and ultimately increased DC and T cell infiltration in tumor deposits^[78-83]^. Other commonly used cytotoxic therapies in sarcomas, including gemcitabine, trabectedin, and eribulin, also have pro-immunogenic effects. Gemcitabine suppresses MDSCs within other cancer subtypes^[84]^, trabectedin has been shown to diminish the production of immunosuppressive cytokines in myxoid liposarcomas^[85]^, and eribulin helps to remodel aberrant tumor blood vessels in murine mouse models^[86]^. In hopes of providing a source of immunogenic neoantigens and pro-inflammatory mediators for poorly immunogenic sarcomas, multiple studies exploring chemotherapy with checkpoint inhibitors have been recently reported or are ongoing.

Two studies have recently been published reporting very promising activity of combination doxorubicin with pembrolizumab. Pollack *et al.*^[32]^ reported a median PFS of 8.1 months in 37 patients with bone and soft tissue sarcomas, with a 73% progression-free rate at 24 weeks, despite an objective response rate of only 19%. Limited correlative data were reported with this study, but suggested that the presence of TILs was associated with inferior PFS. No additional phenotyping was reported to explain this surprising finding. More recently, Livingston *et al.*^[87] ^reported an overall response rate of 36.7%, median PFS of 5.7 months, and a 6 month PFS rate of 44%, in 30 patients with advanced soft tissue sarcomas. Interestingly, analysis of PD-L1 expression in 29 of the 30 patients revealed an association of high PD-L1 expression with objective response rate, however, did not correlate with PFS or overall survival. The presence of TILs was not associated with any clinical outcomes, however, no phenotyping was reported with this finding. Ongoing clinical trials are exploring additional doxorubicin combinations, including doxorubicin with ifosfamide and sintilimab (anti-PD-1, NCT04356872), liposomal doxorubicin with ifosfamide and camrelizumab (anti-PD-1, NCT04606108), and doxorubicin with balstimilab (anti-PD-1) and zalifrelimab (anti-CTLA4, NCT04028063).

Regarding other cytotoxics, multiple studies are evaluating gemcitabine with checkpoint inhibitors for sarcomas (NCT04577014, NCT03123276), as well as a study combining metronomic gemcitabine with doxorubicin, docetaxel, and nivolumab (NCT04535713). Results of a study combining first-line trabectedin, ipilimumab, and nivolumab have been reported, with a best overall response rate of 22%, and median PFS had not been reached at the time of the presentation (NCT 03138161)^[88]^. Finally, an ongoing study of eribulin plus pembrolizumab has shown modest responses in leiomyosarcoma^[89]^, with the full study still pending results (NCT03899805).

Overall, the combination of chemotherapy with checkpoint inhibitors appears the most promising strategy to date to reverse innate immune resistance of most adult sarcomas. However, correlative analysis of these studies to further confirm underlying biomarkers of response and resistance is still in infancy, with many unanswered questions.

### CONCLUSION

In summary, there is still a tremendous amount to learn about resistance to immune therapy in sarcomas, but early studies have shown conclusively that for a subset of patients, immune therapy, including checkpoint inhibitors or adoptive cellular therapies, can be highly effective. Emerging research in looking at the underlying genetic and immune environments of sarcomas suggests that the pivotal mechanism of resistance is a lack of underlying tumor immunogenicity. Based on our prior experience, it appears unlikely that a single agent in combination with immune agents will be sufficient to reverse these hurdles for all sarcomas, but the promising activity of cytotoxic chemotherapy and potentially radiation combinations may eventually emerge as a crucial part of our therapeutic armamentarium. It remains critical that all early phase clinical trials of immune therapy include correlative studies, to ensure that we are learning the most from every patient, and to pool these data to understand mechanisms of response and resistance, to inform the next generation of clinical trials.

## DECLARATIONS

### Copyright

© The Author(s) 2022.
